# Continuing efforts to characterize and treat extrasystoles originating from the left ventricle

**DOI:** 10.1111/anec.13003

**Published:** 2022-09-19

**Authors:** Sinan S. Tankut, David T. Huang

**Affiliations:** ^1^ Division of Cardiology University of Rochester Medical Center Rochester New York USA

It has been 14 years since Doppalapudi et al. first described the distinct clinical syndrome of ventricular tachycardia (VT) originating from the base of the posterior papillary muscle (PPM) in the left ventricle. The characteristics and behavior of VT originating from the PPM were unique and presented several challenges with management, especially with catheter ablations. Seven patients underwent successful RF ablation in the Doppalapudi et al. (2008) study. A mean follow‐up of 9 months demonstrated all patients were free from ventricular arrhythmias (VA) or PVCs. The identification of a VA specific to the papillary muscles led to subsequent studies, each presenting its own challenges.

In the study by Liu et al. electrophysiology studies and radiofrequency ablations were conducted on 424 patients. Data was presented on 27 patients with PVC/VA originating from the left posterior papillary muscle (LPPM) and compared to 21 patients with ventricular arrhythmias originating from the left posterior “branch” or fascicle (LPF) of the Purkinje system. The authors successfully utilized intracardiac ultrasound (ICE) and electroanatomical (EAM) mapping to construct three‐dimensional right and left ventricular models, which should be regarded as standard of care in these patients when catheter‐based intervention is pursued. Several ECG characteristics were retrospectively analyzed and identified to help differentiate the two VAs. In general, lead V1 was consistent with a rSr or R type pattern (left “rabbit ear”) among cases originating from the LPPM. In comparison, V1 lead pattern was mostly consistent with rSr (right “rabbit ear”) among patients with VA originating from the LPF. This may be an expected finding as the origin from the fascicular conduction tissue would resemble a “typical” right bundle branch “block” pattern, rather than an “atypical” one with ectopy originating from papillary muscle, a myocardial source. Several further differentiating ECG characteristics were mentioned throughout the study. Excitation mapping was utilized to identify the earliest site of VA/PVC activation, and again intracardiac ultrasound and EAM were utilized to recognize target ablation sites. After a 24‐month follow‐up period, 26/27 patients had no primary symptoms or VA, and one patient experienced PVCs with differing ECG morphology. (Liu et al., 2022)

This study supports the need for the utilization of both ultrasound and EAM to create three‐dimensional cardiac structures to achieve further ablation success. Proietti et al. presented one of the first case series to demonstrate both safety and effectiveness of left ventricular PM VA ablation using ICE for three‐dimensional EAM. (Proietti et al., 2017) Since, the integration of ICE and EAM has become increasingly more utilized. The 2019 APHRS consensus statement in collaboration with HRS/EHRRA/LAHRS gives the utilization of 3D mapping with detailed activation mapping and anatomical representation a class I recommendation for idiopathic, nonoutflow tract ventricular tachycardia ablations. (Kim et al., 2020) Therefore, it is no surprise that the authors achieved success with the use of these now standard intraprocedural tools. As mentioned in their paper, the anatomy of the papillary muscle along with catheter‐tissue contact was delineated with ICE imaging, further enhancing their ablation success. (Liu et al., 2022)

Liu et al. provide certain ECG characteristics that are unique to arrhythmias originating from LPPM and LPF. While there are certain generalizations for differentiating the two arrhythmias, (such as lead V1 finding),Kim et al., 2020 the detailed ECG descriptions for both arrhythmias were cumbersome. Localization of VA or PVCs using certain ECG criteria is crucial for preprocedural planning; however, pinpointing certain cardiac structures to various ECG findings can be difficult. Furthermore, providing detailed ECG findings has its pitfalls when the relative positioning and angulation/rotation of these cardiac structures, along with inconsistent lead placement can result in variability of QRS morphologies.

The target ablation site was determined by identifying the earliest activation point during excitation mapping. This was difficult to accomplish due to the varying morphology of the papillary muscle and required detailed mapping. In this series, the effective target ablation point was mostly found in the middle of the papillary muscle (20/36 patients). (Liu et al., 2022) However, it is important to note the limitations of activation mapping for nonfocal, re‐entrant circuits. Just as with the observed variations in papillary muscle anatomy, there is described variability in the left ventricular Purkinje network system, particularly in relation to septal branches. (Demoulin & Kulbertus, 1972) In a re‐entrant circuit, there are prolonged areas of slow conduction with some beats that can appear presystolic. Therefore, utilization of activation mapping alone without the use of concurrent entrainment mapping can result in shifting of the exit site without disruption of the critical isthmus. (Guandalini et al., 2019; Noheria et al., 2015)

Finally, the study shows excellent results on postprocedural PVC/VA burden with assessment of noncontinuous cardiac monitors. We would have liked to see similar results with extensive, continuous monitoring as patients can be asymptomatic with these VA and/or PVCs. With improvements in wearable monitors and implantable cardiac devices, we believe that arrhythmia burden evaluation should be conducted via continuous monitors with any study moving forward.

Liu et al. provide a supportive research study that emphasizes the need for integrating ICE and EAM to enhance ablation success from the LPPM. Moving forward, we anticipate three‐dimensional EAM to become standard of care for nonoutflow, idiopathic VA, and/or PVC ablations.

## CONFLICT OF INTEREST

Fellowship support: Medtronic, Boston Scientific, Abbott; Research support: Biosense Webster, Biotronik, and Medtronic. Consulting arrangement: Boston Scientific.
